# The effect of written information and counselling by an advanced practice nurse on resilience in women with vulvar neoplasia six months after surgical treatment and the influence of social support, recurrence, and age: a secondary analysis of a multicenter randomized controlled trial, WOMAN-PRO II

**DOI:** 10.1186/s12905-020-00965-z

**Published:** 2020-05-06

**Authors:** Sabine Kofler, Andrea Kobleder, Stefan Ott, Beate Senn

**Affiliations:** 1 Institute for Applied Nursing Sciences IPW-FHS, FHS St. Gallen, University of Applied Sciences, Rosenbergstrasse 59, 9001 St. Gallen, Switzerland; 2 Lecturer for Business Mathematics and Statistics, University of Applied Sciences FHS St. Gallen, Rosenbergstrasse 59, 9001 St. Gallen, Switzerland; 3 Sydney Nursing School, The University of Sydney, Mallett Street 88, 2050 Camperdown, NSW Australia

**Keywords:** Resilience, Vulvar neoplasia, Surgical treatment, Influencing factors, APN

## Abstract

**Background:**

Women with vulvar neoplasia often complain about physical and psychological distress after surgical treatment. Lack of information and support can influence resilience. Whether an information-related intervention through an advanced practice nurse supports resilience and which other factors affect resilience in women with vulvar neoplasia has never been investigated.

**Methods:**

The aims of this study were (a) to analyse whether counselling based on the WOMAN-PRO II program causes a significant improvement in the resilience scores of women with vulvar neoplasia compared to written information and (b) to identify the potential predictors of resilience.

A randomized controlled trial was conducted in women with vulvar neoplasia (*n* = 49) 6 months after surgical treatment in four Swiss hospitals and one Austrian hospital. Analyses of resilience and its predictors were performed using a linear mixed model.

**Results:**

Thirty-six women (intervention I, *n* = 8; intervention II, *n* = 28) completed the randomized controlled trial. In total, 13 women (26.5%) dropped out of the trial. The resilience score did not differ significantly between the two interventions three and six months after randomisation (*p* = 0.759). Age (b = .04, *p* = 0.001), social support (b = .28, *p* = 0.009), counselling time (b = .03, *p* = 0.018) and local recurrence (b = −.56, p = 0.009) were identified as significant predictors of resilience in the linear mixed model analyses.

**Conclusion:**

The results indicate that the WOMAN-PRO II program as single intervention does not cause a significant change in the resilience scores of women with vulvar neoplasia 6 months after surgery. Predictors that promote or minimise resilience have been identified and should be considered when developing resilience programs for women with vulvar neoplasia. A repetition of the study with a larger sample size is recommended.

**Trial registration:**

The WOMAN-PRO II program was registered in ClinicalTrials.gov NCT01986725 on 18 November 2013.

## Background

Vulvar neoplasia includes vulvar intraepithelial neoplasia as a precancerous cellular change as well as vulvar cancer as a cancerous cellular change in the external female genitalia [1]. Although vulvar neoplasia is a rare disease, its incidence has increased globally over the past decade, especially among younger women [2]. In Germany, the incidence of vulvar cancer increased from 1.6 cases per 100,000 women annually during 1974–1978 to 7.9 cases per 100,000 women annually during 2009–2013 [3].

Surgical treatment is considered the standard intervention for vulvar neoplasia [4–6]. Despite surgical advances, women in the post-surgical phase experience physical and psychosocial symptoms, including pain, fatigue and altered self-image, in addition to emotional and interpersonal distress or uncertainty [7, 8]. Additionally, vulvar neoplasia remains a stigmatised condition associated with poor hygiene or promiscuity [8]. Hence, affected women feel isolated and are often unable to speak about their condition [9, 10].

It is well established that cancer treatment and the accompanying symptom-related distress cause significant adversity, which can lead to negative outcomes, such as post-traumatic stress disorder, depression or anxiety [11, 12].

Studies describe resilience as an important factor for overcoming such adversities. Resilience is a multidimensional concept, defined in different ways. We interpret that resilience is a capacity and dynamic process to successfully cope with a significant change or adversity and it can be facilitated through interventions [13, 14]. Important elements related to the concept of resilience in cancer patients include confidence, self-transcendence of the cancer experience and self-esteem. Confidence is characterised by the perception that an individual has control over environmental circumstances [15]. Under adverse circumstances, such as during cancer treatment and symptom-related distress, uncertainty can lead to loss of control. Haase [15] states that uncertainty arises when people lack sufficient information about the treatment or do not understand the information they receive.

While symptom-related distress and uncertainty vary with cancer type and treatment, they are particularly pronounced in cancers associated with taboos and stigmatisation [7, 16]. Moreover, phenomenological studies have shown that women with vulvar neoplasia experience a general lack of information about symptom management and strategies to handle their situation [8–10].

Due to the increasing number of mental disorders associated with cancer and cancer treatment, the concept of resilience is becoming increasingly important in cancer care [14, 17]. Nurses are in a crucial position to facilitate resilience [18]. With growing interest, it is important to understand how nurses can support women with vulvar neoplasia and help them adapt to adverse circumstances. So far, few intervention studies have examined the effect on resilience [14, 17, 19]. These studies have mainly been psychotherapeutically oriented [17]. Whether nurse-led information-related interventions affect resilience in women with vulvar neoplasia and what other aspects contribute to resilience in this population remain unknown. Therefore, the aim of the present study was (a) to investigate the effect of systematic counselling by an advanced practice nurse (APN) compared to written information on resilience in women with vulvar neoplasia 6 months after surgery and (b) to examine the influence of social support, age, counselling time and local recurrence on resilience in this population.

## Methods

### Study design

This study is a secondary analysis of quantitative data from the mixed methods project WOMAN-PRO II (clinical trial ID: NCT01986725). The quantitative part of the WOMAN-PRO II study was a two-arm, multicenter, randomized controlled, parallel-group, phase II trial with two interventions and repeated measures during 6 months. The primary outcome was the symptom prevalence in women with vulvar neoplasia over 6 months after surgical treatment. Results focusing on this outcome are published elsewhere [20].

In the present secondary analysis, we examined the effect of written information (intervention I) and systematic counselling by an APN (intervention II) on resilience in women with vulvar neoplasia at baseline as well as three and six months after surgery. In addition, we tested whether social support, age, counselling time and local recurrence have an influence on resilience in women with vulva neoplasia.

### Interventions

The contents of the two interventions are described in Table 1. Participants in intervention I received standard care and written information, while those in intervention II additionally received systematic counselling by an APN with a focus on promoting the self-management of post-surgical symptoms. In this program, women with vulvar neoplasia are taught to use a symptom diary in order to help them recognise and assess their symptoms at different levels, e.g. wound-related symptoms, difficulties, feelings, thoughts and behaviours. The assessments of symptoms and distress are discussed in the APN consultation, focusing on tailored information, motivational interviewing, self-management and behavioural change. A detailed description of the interventions can be found in a previous study [20].

**Table 1:** Description of the two interventions arms

	**Written information (intervention I)**	**Counselling based on the WOMAN-PRO II program (intervention II)**
*Intervention*	- Standard care and written information	- Intervention I and counselling by an APN or nurse with equivalent education
*Mode of delivery*	- Face-to-face	- Face-to-face and via a phone call
*Content*	Set of leaflets, including the following: - wound care after discharge - healthcare services in the hospital and community (e.g. by a psycho-oncologist)	Nurse-led follow-up consultations, including the following: - symptom self-assessment with the WOMAN-PRO symptom diary [21] (based on four domains: wound-related symptoms, psychosocial symptoms, difficulties in daily life and information needs) - healthcare services - decision-making
*Based on*	- Standard care: local standards and existing guidelines [2, 22] - Written information: developed by a group of clinical experts	- Evidence-based counselling guideline [23]
*Intensity*	- 10 to 30 min - During hospitalisation and routine follow-up (three and six months after discharge)	- 10 to 50 min - One week after surgery, two weeks after discharge, three months after surgery and six months after surgery
*Implementation*		- Training (12 h) - Opportunity to receive supervision during the study

APN = advanced practice nurse

### Sample and setting

Data were collected from September 2013 until May 2015 from four Swiss hospitals (two regional and two university hospitals) and one Austrian university hospital.

The sample size calculation was driven by the symptom scores of a previous study [7]. A total sample of 90 women in both the arms was required. An attrition rate of 15% was added; therefore, 106 women needed to be recruited. The randomization ratio was 1:2 (intervention I: *n* = 30; intervention II: *n* = 60, plus 16 to compensate for expected drop-outs) based on the recommendation of Dodd et al. [24], in favour of counselling at the expense of written information.

Women aged over 18 years who were diagnosed with vulvar intraepithelial neoplasia or vulvar cancer and who underwent planned surgical treatment in one of the designated hospitals were included in the study. Women who were terminally ill or not able to complete the questionnaire for cognitive, linguistic, emotional or physical reasons were excluded.

### Randomization

A nurse or physician in the ambulatory setting or on the ward invited eligible women to participate in the study. A computer-generated block randomization list with one stratification factor (vulvar intraepithelial neoplasia or vulvar cancer) was generated. After having signed the informed consent, the eligible women were randomly assigned to intervention I or II. Sequentially numbered, opaque, sealed envelopes were used to guarantee allocation concealment. The envelopes were prepared by persons not involved in the study. To determine group allocation, the APN opened the envelopes in numerical order in front of the patient and assigned the patient to intervention I or II.

Data focusing on resilience and social support were collected at three time points: at diagnosis (t0), 3 months after surgery (t3) and 6 months after surgery (t4). Consultation took place at five time points, beginning at diagnosis (t0) and ending 6 months (t4) after surgery. Medical data, e.g. local recurrence, were collected 7 days after surgery (t1), and sociodemographic data were collected once at t0.

Owing to the nature of the intervention, blinding of the participants was not possible. However, the participating women and healthcare staff were blinded to group assignments and differences between the two interventions. Additionally, external quality audits based on the guidelines of the National Cancer Institute were conducted in both the intervention arms [25]. The aim of the audit was to review (1) the study progress, (2) adherence to the study protocol, (3) the APN’s counselling knowledge and (4) case reports of randomly selected study records.

### Measurements

#### Sociodemographic and medical data

Sociodemographic data were collected using an 11-item scale concerning age, marital status, number of children, education, employment status, living situation, post-surgical wound management at home and health insurance. Medical data were collected using a 16-item form concerning diagnosis, cancer stage, initial treatment and wound treatment. Both the questionnaires were developed for the WOMAN-PRO II study and tested previously [7].

#### Connor–Davidson resilience scale (CD-RISC)

Resilience was assessed using the author-approved German translation of the 10-item scale, CD-RISC 10. This unidimensional instrument reflects the ability to bounce back from various challenges, such as illness, emotional pressure or painful feelings. Items are rated on a 5-point scale (0 = not true at all to 4 = true nearly all the time), providing a total sum score ranging from 0 to 40, with higher scores reflecting greater resilience [26]. The German translation of CD-RISC 10 has acceptable psychometric properties with high internal consistency (Cronbach’s alpha = 0.84) [27]. The internal consistency of CD-RISC 10 at baseline was very high in the present study (α_t0_ = 0.89).

#### Multidimensional scale of perceived social support (MSPSS)

Social support was assessed using the German version of the 12-item MSPSS [28]. This instrument measures the perceived adequacy of social support from family, friends and significant others. These aspects form three subscales with four items each. Items are rated on a 7-point scale (1 = very strongly disagree to 7 = very strongly agree). Higher scores on each subscale indicate higher levels of perceived support. The mean of the three subscales reflects global satisfaction with perceived social support. The internal consistency of the original scale is high (Cronbach’s alpha = 0.88) and its construct validity is adequate [28]. The internal consistency of MSPSS at baseline was very high in the present study (α_t0_ = 0.86).

### Counselling time

The invested counselling time was listed by the APN after each consultation.

### Statistical analysis

Descriptive statistics (frequencies, median [Mdn] and interquartile range [IQR]) were used to characterise the basic properties of our sample. Group differences in sociodemographic and medical data at baseline were assessed using non-parametric tests (Mann–Whitney U test and Fisher’s exact test).

Mean scores were calculated for CD-RISC and MSPSS. Global score of MSPSS was used for the analysis. Furthermore, linear mixed models were used to determine differences between the intervention arms across the time points and to assess the impact of other factors, such as age, social support, counselling time and local recurrence, on resilience. Step by step, variables were integrated into the model based on a significant change in − 2 log likelihood (−2LL). Apart from the main effects, significant interactions were also investigated; a significant change was not noticed over time. Therefore, it was not necessary to adapt the covariance structure. Based on graphical techniques (residuals plots and Q–Q plots) and appropriate tests (the Kolmogorov–Smirnov test), we ensured the assumptions of the linear mixed models to hold.

A two-tailed *p*-value of 0.05 was considered to indicate statistical significance. Statistical analyses were performed using IBM SPSS Statistics for Windows (version 23.0) based on the intention-to-treat principle.

## Results

Of the 93 eligible patients, 49 were included in the analysis. Forty-four eligible women refused to participate because of feeling overburdened, time constraints, missing trust or loss of interest in the research. During the follow-up phase, a total of 13 women (26.5%) dropped out, primarily because they were no longer available after being transferred to another hospital. Participants who dropped out did not differ significantly in sample characteristics or resilience scores by group or compared to those who completed the study. The process of patient recruitment, randomization and follow-up is presented in the publication of the primary outcomes [20].

The sociodemographic characteristics of the sample are shown in Table 2. The participants’ age ranged from 24 to 81 years (Mdn = 57.0; IQR = 47.0; 66.0*)*. Thirty-one women (63.3%) had a vulvar cancer diagnosis and nine women (18.4%) had a local recurrence. Of the 49 women, 21 (42.9%) were married and 33 (67.3%) had 10 or fewer school years. No significant difference could be observed between the two intervention arms in terms of demographic or clinical characteristics at baseline.

**Table 2:** Sample characteristics at baseline by intervention arms

**Characteristics**	**Written information (*n*** **= 13)**	**counselling (*n*** **= 36)**	**P**	**Total**
Number of patients per country; n			.53^a^	
Austria	7	15		22
Switzerland	6	21		27
Age (years); Mdn (IQR)	57.0 (45.0; 69.0)	56.5 (45.5; 64.5)	.62^b^	57.0 (47.0; 66.0)
Missing; n (%)	0 (0.0)	6 (16.7)		6 (12.2)
Marital status; n			.19^a^	
Married	8	13		21
Single/widowed/divorced	5	22		27
Missing; n (%)	0	1 (2.8)		1 (2.0)
Children			.28^a^	
Having at least one child; n	10	20		30
Having no children	2	13		15
Missing; n (%)	1 (7.7)	3 (8.3)		4 (8.2)
Educational level; n			.52^b^	
10 or fewer school years	8	25		33
> 10 years	5	10		15
Missing; n (%)	0 (0.0)	1 (2.8)		1 (2.0)
Local recurrence, n			1.00^a^	
Yes	3	6		9
No	9	22		31
Missing; n (%)	1 (7.7)	8 (22.2)		9 (18.4)
Type of vulvar disease			.74^a^	
Vulvar intraepithelial neoplasia	4	14		18
Vulvar squamous cell carcinoma	9	22		31
Missing; n (%)	0 (0.0)	0 (0.0)		0.(0.0)
Counselling time (minutes); Mdn (IQR)	―	30.0 (20.0; 45.00)	―	30.0 (20.0; 45.00)
Missing; n (%)	―	9 (25.0)		9 (25.0)
MSPSS mean score^c^; Mdn (IQR)	6.6 (6.3; 6.9)	6.7 (6.0; 7.0)	.95^b^	6.6 (6.1; 7.0)
Missing; n (%)	3 (23.1)	8 (22.2)		11 (22.4)
Resilience mean score^d^; Mdn (IQR)	3.4 (2.5; 3.8)	3.2 (2.7; 3.7)	.51^b^	3.3 (2.6; 3.7)
Missing; n (%)	3 (23.1)	8 (22.2)		11 (22.4)

Mdn = median; IQR = interquartile range (1. quartile; 3. quartile); MSPSS = Multidimensional Scale of Perceived Social Support; p = p*-*value

^a^Fisher’s exact test

^b^Mann–Whitney U test

^c^Likert scale ranges from 1 to 7; high scores indicate higher levels of perceived social support

^d^Likert scale ranges from 0 to 4; higher values higher mean resilience

**Fig. 1:**
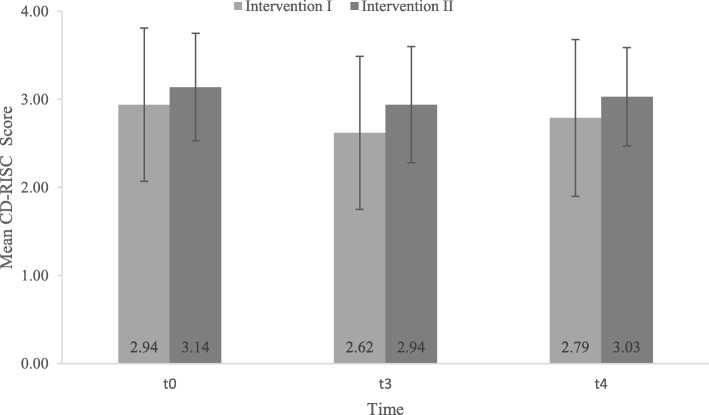
Resilience scores (mean; standard deviation) of the two intervention arms at the three time points

The linear mixed model showed no significant difference in resilience between the two intervention arms (*p* = 0.759). In addition, the scores were not statistically different between the intervention arms at the three time points (*p* = 0.345). The course of resilience over the three time points in both the intervention arms is displayed in Fig. 1.

The results of the linear mixed model indicated a significant impact of local recurrence (*p* = 0.009), age (*p* = 0.001), social support (p = 0.009) and counselling time (*p* = 0.018) on resilience. While age, social support (MSPSS) and counselling time had a positive influence, local recurrence had a negative impact on resilience. The joint influence of age and counselling time was significant as well (*p* = 0.006), with the impact of counselling time decreasing with increasing age. The details are shown in Table 3.

**Table 3:** Linear mixed model analyses predicting the change in resilience

	b	t	p	95% CI
Local recurrence (0 = no, 1 = yes)	−.56	−2.70	.009	−.98, −.14
Age	.04	3.40	.001	.01, .06
Counselling time (minutes)	.03	2.44	.018	.01, .06
Social support	.28	2.72	.009	.07, .49
Time (for 6 month)	−.09	−.95	.345	−.27, .10
Age × Counselling time	−.0008	−2.88	.006	−.001, −.002

95% CI = 95% confidence interval

## Discussion

The aim of this study was to evaluate the effect of written information and counselling by an APN on resilience in women with vulvar neoplasia three and six months after surgery. Additionally, we investigated the influence of social support, age, counselling time and local recurrence on resilience in this population. The results showed no significant difference in resilience between the two intervention arms. Furthermore, the findings indicated that social support, longer counselling time and higher age were associated with a higher resilience score, whereas local recurrence was associated with a lower resilience score. The six-month period was found to have no influence on resilience.

To our knowledge, this is the first study to explore the effect of information-related interventions on resilience in women with vulvar neoplasia. One explanation for the non-significant results could be that the benefit of the intervention could be noticeable at a later time and more time could be needed for adaptation, probably due to the dynamic process of resilience [14]. A study of a recently published review investigated data over an average five-year period [29].

The results may indicate that a single intervention, such as counselling by an APN to improve symptom management, is not sufficient to promote resilience. This would be in contrast to the conclusion of Haase [15], who suggested that specific factors (e.g. illness-related factors) can be used to promote resilience. This implies that multimodal interventions are necessary to facilitate resilience in cancer care. A possible multimodal intervention could be based on the adolescent resilience model by Haase [15]. The theory involves various factors, such as illness-related, family, social and individual factors. This theory could be particularly interesting for younger women with vulvar neoplasia. A more holistic model is the society-to-cells model described by Szanton et al. [18]. This model includes factors at the cellular, physiological, individual, family and society levels and should be particularly used for nursing intervention to facilitate resilience.

The results could also indicate that the intervention period should be extended, i.e. counselling should offered directly after the diagnosis. A recent review revealed that interventions provided immediately after the diagnosis and in parallel with somatic treatment had the greatest effect on resilience [17]. Therefore, a multiprofessional approach should be considered. Adequate information about diagnosis and treatment, e.g. fertility preservation or preservation of sexual function, seems to be as important as the treatment of symptoms post-operatively in women with vulvar neoplasia [30, 31]. Various reviews have stated that an individualised approach should be used for surgical treatment, especially in patients with advanced disease. Additionally, more conservative and less radical surgery and the new reconstructive plastic techniques have been associated with lesser physical and psychological morbidity [5, 6].

The results of the linear mixed models reveal that resilience was positively associated with age. Previous studies on individuals with colorectal cancer and the general population support our result that older age is associated with higher resilience [32, 33].

One explanation of this result is that older women have already completed family planning or can look back on a fulfilled life. Thus, they probably have less emotional distress and can better accept the situation [32]. For women of childbearing age, the fact that they cannot have children may be worse than the diagnosis itself [30]. This can lead to an additional stressor in younger women, and more time and support would therefore be needed for successful adjustment. Therefore, it should be noted that younger women show more psychosocial symptoms than older women [7].

The present study revealed that resilience is positively associated with a longer counselling time. This result is in line with the finding by Ludolph et al. [17]. They found, in a review that the greatest effect strength is achieved with a longer intervention duration, i.e. more than 12 sessions and a cumulative duration of at least 24 h.

The WOMAN PRO program was based on the self-management concept. One of five core-self-management skills is problem solving. Here, the problem defined by the patient is considered specifically, and the patient is guided to find the most suitable solution [34]. Therefore, it can be assumed that a longer counselling time contributes to resolving disease-related uncertainties and developing effective problem-solving strategies [16] and supports to cope successfully with symptom-related distress. This assumption is confirmed by a qualitative study, where women reported that they felt safe and secure because they were more confident after the APN actively took time to talk to them and discuss their concerns [35].

At this point it should be emphasized that regular quality controls were carried out for this study. The result can be an indication of appropriate quality of counselling and standard of care.

Interestingly, our results indicated that the impact of counselling time decreases with increasing age. Older individuals have possibly been confronted with other adverse circumstances (e.g. previous illnesses, divorce or the death of a loved one), resulting in positive problem-solving strategies [36]; hence, lesser support or information may be required. On the other hand, older women may have more difficulty talking about their symptoms. A previous study mentions that older women are particularly ashamed to talk about their disease [10].

We found that a higher level of perceived social support predicts higher resilience. This result is not surprising; previous studies confirm the buffering effect of social support [37, 38]. Qualitative studies have indicated that social support, especially for patients with taboo diseases, such as vulvar neoplasia, is important to successfully cope with the situation. It allows talking about the experience and contributes to reduced stress and higher emotional well-being [35, 39].

Social support can be from different sources, such as family, friends or other important persons [40]. APNs or nurses can play an important role in encouraging women to speak about cancer, request assistance or minimise prejudices. Kobleder et al. [35] have described that women felt more secure and less alone in the experience of their illness after having the possibility of contacting an APN.

Finally, the present findings reveal that resilience is negatively correlated with recurrent disease. In a descriptive study, Dubey et al. [41] showed that resilience in patients with different types of cancer does not correlate with recurrence. Recurrence possibly promotes hopelessness and leads to lower resilience [42] as well as fear and uncertainty about the further course of the disease [43].

### Limitations

This study has several limitations. First, the reduced sample size may lead to a loss of accuracy and, under certain circumstances, statistical significance. Additionally, the described drop-out rate could have an impact on the validity of the study, however, participants who dropped out did not differ significantly compared to those who completed the study. Extended data collection was not possible because of the limited study resources.

Second, this study did not account for potential confounders, such as time since the cancer diagnosis or other sociodemographic variables (e.g. education and marriage). Further, some participants might have experienced other types of traumatic events that were not assessed.

Third, participants with mental disorders were excluded, thereby increasing the risk of bias, as resilience correlates with different mental disorders, e.g. depression or anxiety [26].

Finally, the short duration of the study did not allow for assessing the long-term effect of the intervention on the development of resilience.

## Conclusions

In conclusion, our results indicate that the WOMAN-PRO II program should be further developed to promote resilience and to support women with vulvar neoplasia for facing cancer-related distress. Furthermore, perceived social support, particularly in the form of communication, it seems important for developing resilience in women with vulvar neoplasia. This might be considered with regard to further development of the intervention, e.g. by increasing the intensity of consultations with a prolonged counselling time or active involvement of family members. Moreover, age and individual experience should be taken into consideration. This means that younger women and persons who and people who experience a recurrence of the disease need more intervention.

In a further study, the negative relationship between resilience and recurrence should be explored in women with vulvar neoplasia in order to detect unmet supportive care needs and understand the related dynamic of resilience.

With regard to written information, no implications for clinical practice can be derived on the basis of these results. Predictors indicate that the implementation of counselling may be useful to promote resilience in women with vulvar neoplasia. Further research is needed to validate this hypothesis. To achieve a larger sample size, more hospitals should participate. Moreover, we recommend recruiting the calculated sample size (*n* = 106). Therefore, the recruitment phase should be extended. Finally, due to the dynamic process of resilience, the follow-up phase should be prolonged to at least one year.

## Data Availability

The data that support the findings of this study and the trial protocol are available from the corresponding author (Sabine Kofler) upon reasonable request.

## Abbreviations

APN: Advanced practice nurse
CD-RISC: Connor–Davidson Resilience Scale ()
MSPSS: Multidimensional Scale of Perceived Social Support ()
Mdn: Median
IQR: Interquartile range
